# Neutrophil-to-lymphocyte ratio is a prognostic marker in bladder cancer patients after radical cystectomy

**DOI:** 10.1186/s12885-016-2219-z

**Published:** 2016-03-05

**Authors:** Takashi Kawahara, Kazuhiro Furuya, Manami Nakamura, Kentaro Sakamaki, Kimito Osaka, Hiroki Ito, Yusuke Ito, Koji Izumi, Shinji Ohtake, Yasuhide Miyoshi, Kazuhide Makiyama, Noboru Nakaigawa, Takeharu Yamanaka, Hiroshi Miyamoto, Masahiro Yao, Hiroji Uemura

**Affiliations:** Department of Urology, Graduate School of Medicine, Yokohama City University, Yokohama, Japan; Departments of Urology and Renal Transportation, Yokohama City University Medical Center, Yokohama, Japan; Department of Biostatistics, Yokohama City University Graduate School of Medicine, Yokohama, Japan; Departments of Pathology and Urology, Johns Hopkins University School of Medicine, Baltimore, USA

**Keywords:** Bladder cancer, Radical cystectomy, Biomarker, Neutrophil-to-lymphocyte ratio

## Abstract

**Background:**

There is no reliable biomarker for predicting the prognosis of patients who undergo radical cystectomy for bladder cancer. Recent studies have shown that the neutrophil-to-lymphocyte ratio (NLR) could function as a useful prognostic factor in several types of malignancies. This study aimed to assess the usefulness of NLR in bladder cancer.

**Methods:**

A total of 74 patients who underwent radical cystectomy in our institutions from 1999 to 2014 were analyzed. The NLR was calculated using the patients’ neutrophil and lymphocyte counts before radical cystectomy. An immunohistochemical analysis was also performed to detect tumor infiltrating neutrophils (CD66b) and lymphocytes (CD8) in bladder cancer specimens.

**Results:**

A univariate analysis showed that the patients with a high NLR (≥2.38; HR = 4.84; *p* = 0.007), high C-reactive protein level (>0.08; HR = 10.06; *p* = 0.030), or pathological lymph node metastasis (HR = 4.73; *p* = 0.030) had a significantly higher risk of cancer-specific mortality. Kaplan-Meier and log-rank tests further revealed that NLR was strongly correlated with overall survival (*p* = 0.018), but not progression-free survival (*p* = 0.137). In a multivariate analysis, all of these were found to be independent risk factors (HR = 4.62, 10.8, and 12.35, respectively). The number of CD8-positive lymphocytes was significantly increased in high-grade (*p* = 0.001) and muscle-invasive (*p* = 0.012) tumors, in comparison to low-grade and non-muscle-invasive tumors, respectively.

**Conclusions:**

The NLR predicted the prognosis of patients who underwent radical cystectomy and might therefore function as a reliable biomarker in cases of invasive bladder cancer.

## Background

Urinary bladder cancer is one of the most commonly diagnosed malignancies [1]. Two-thirds to three-fourths of patients with bladder tumor initially present with non-muscle-invasive (pTa or pT1) disease that can often be treated with conservative approaches, however, many patients suffer from recurrence, occasionally with grade and/or stage progression. In contrast, for patients with muscle-invasive bladder cancer the gold standard treatment is radical cystectomy. Radical cystectomy is also occasionally performed in patients with stage Ta-1 or Tis tumors that are resistant to intravesical instillation therapy [2]. Though radical cystectomy has some therapeutic benefits, it is a potentially invasive surgery which requires urinary diversion. Thus, a new marker to predict the prognosis is needed to determine whether patients should receive radical cystectomy.

Previous reports have indicated a variety of tissue- or urine-based biomarkers that can be used for predicting the recurrence and progression of bladder cancer; these have often been PCR- or immunohistochemistry-based methods [3]. A simple, inexpensive and highly accurate method would be preferable for daily clinical use. The neutrophil-to-lymphocyte ratio (NLR), which can be easily calculated from routine complete blood counts (CBCs) in peripheral blood samples has been suggested as a predictor, not only for the systemic inflammatory response in critical care patients [4] but also for the prognosis of some solid malignancies including bladder cancer [2, 5–13]. Additionally, the NLR can be obtained retrospectively even in the postoperative follow-up because CBCs are routinely examined in various stages of bladder cancer therapy. In the current study, we assessed the utility of the NLR in the prediction of the prognosis of patients with bladder cancer who underwent radical cystectomy.

## Methods

### Patients

A total of 74 patients underwent radical cystectomy for bladder cancer in Yokohama City University Hospital (Yokohama, Japan) from February 1999 to April 2014. All patients were Japanese. Written informed consent was obtained from each patient, and the institutional review board of the hospital approved this study. The patients were followed up every three months for two years postoeratively and then every 6 months thereafter by either cystoscopy or CT.

### Clinical and laboratory assessments

The NLR was calculated using neutrophil and lymphocyte counts via CBCs that were obtained a few days before surgery. CBCs include the number of blood cells, platelets, and white blood cells with differentiation. Other variables, including C-reactive protein (CRP) and lactate dehydrogenase (LDH), were simultaneously obtained. We determined the cut-off points of the NLR, CRP, and LDH, based on the sensitivity and specificity levels derived from area under receiver operator characteristics (AUROC) curves plotted for disease progression or death. None of the patients demonstrated either systemic inflammation or blood disease at the time of the blood examinations.

### Immunohistochemistry

A bladder cancer tissue microarray (TMA) was obtained from US Biomax (Rockville, MD, USA). The prognostic data were unavailable for these cases. Immunohistochemical staining was performed for both CD66b and CD8 to detect tumor infiltrating neutrophils and lymphocytes, respectively. An immunohistochemical analysis was performed, as described previously, using a primary antibody to CD66b (clone G10F5, diluted at 1:200, BD Biosciences, San Jose, CA, USA) or CD8 (clone C8/144B, diluted at 1:100, DAKO Corporation, Carpenteria, CA, USA) [14]. The slides were then examined by a single pathologist (HM) blinded to the sample identity. The total number of CD68-positive or CD8-positive cells was counted in each TMA core.

### Statistical analysis

The patients’ characteristics and preoperative factors were analyzed by Mann–Whitney *U* and chi-square tests. Multivariate logistic regression models were used for detecting the individual factors. The Kaplan-Meier product limit estimator was used to estimate progression-free survival (PFS) and overall survival (OS). Survival duration was defined as the time between radical cystectomy and tumor progression or death. The log-rank test was performed for comparison. A *P* value of <0.05 was considered to be statistically significant.

## Results

### Patients’ characteristics

The median and mean (± SD) ages of the 74 patients (male, *n* = 58; female, *n* = 16) were found to be 65 and 64.1 (±9.7) years at 24.2 and 29.6 (±24.2) months of follow-up after radical cystectomy, respectively. The time of diagnosis varied in each patient, however, no differences were observed regarding when the patients received RC. The clinicopathologic data of these patients, such as the ECOG-performance status, the performance of neoadjuvant/adjuvant systemic chemotherapy, the clinical/pathological T stage, the presence of lymph node metastasis, and the surgical margin, are summarized in Table 1. There was no correlation between tumor invasiveness and various factors, including age, sex, and tumor size. To exclude any difference in the diagnostic time, we classified the patients into two groups according to the time of diagnosis and no differences were observed.

**Table 1:** Patient characteristics

	Total (*n* = 74)	NLR < 2.38	NLR ≥ 2.38	*p*-value
Age				
<65 years	32 (43.2 %)	24 (52.9 %)	8 (34.8 %)	0.324
≥65 years	42 (56.8 %)	27 (47.1 %)	15 (65.2 %)	
Gender				
Female	16 (21.6 %)	11 (21.6 %)	5 (21.7 %)	0.987
Male	58 (78.4 %)	40 (78.4 %)	18 (78.3 %)	
ECOG-PS				
0	68 (91.9 %)	48 (94.1 %)	20 (87.0 %)	0.270
≥1	6 (8.1 %)	3 (5.9 %)	3 (13.0 %)	
Neoadjuvant Chemotherapy				
No	64 (86.5 %)	45 (88.2 %)	19 (82.6 %)	0.847
Yes	10 (13.5 %)	6 (11.8 %)	4 (17.4 %)	
Clinical T stage				
≤2	38 (51.4 %)	26 (51.0 %)	12 (52.2 %)	0.924
≥3	36 (48.6 %)	25 (49.0 %)	11 (47.8 %)	
Clinical Lymph Node Metastasis				
No	70 (94.6 %)	48 (94.1 %)	22 (95.7 %)	0.633
Yes	4 (5.4 %)	3 (5.9 %)	1 (4.3 %)	
Surgical Margin				
Negative	68 (91.9 %)	45 (88.2 %)	23 (100.0 %)	0.097
Positive	6 (8.1 %)	6 (11.8 %)	0 (0.0 %)	
Adjuvant chemotherapy				
No	49 (66.2 %)	35 (68.6 %)	14 (60.9 %)	0.514
Yes	25 (33.8 %)	16 (31.4 %)	9 (39.1 %)	
Pathological T stage				
≤2	53 (71.6 %)	37 (72.5 %)	16 (69.6 %)	0.792
≥3	21 (28.4 %)	14 (27.5 %)	7 (30.4 %)	
Pathological Lymph Node Metastasis				
No	64 (86.5 %)	43 (84.3 %)	21 (91.3 %)	0.339
Yes	10 (13.5 %)	8 (15.7 %)	2 (8.7 %)	

*ECOG-PS* Eastern Cooperative Oncology Group performance status

### The NLR cut-off value

**Fig. 1:**
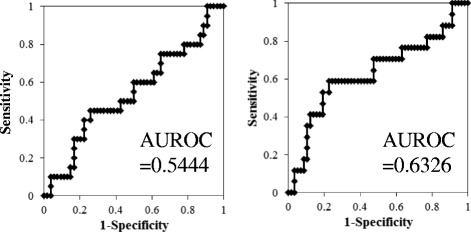
Value of the area under the receiver operating characteristics (AUROC) curve

Based on the AUROC curve, the NLR cut-off point was determined to be 2.38 for both progression and death (AUROC: 0.544 and 0.633, respectively) [Fig. 1]. There were no statistically significant differences in the baseline characteristics of patients with NLRs of <2.38 in comparison to those with NLRs of ≥2.38. Similarly, the cut-off values of CRP (0.08) and LDH (158) were set (data not shown).

### The NLR value and patient outcomes

**Fig. 2:**
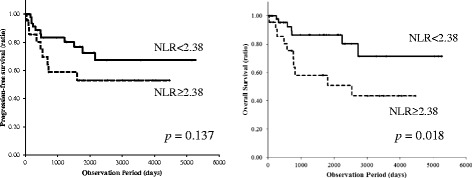
Progression-free and overall survivals according to the NLR

We first performed a univariate analysis to assess the risk of death after radical cystectomy. The NLR (HR = 4.84, *p* = 0.007), CRP (HR = 10.06, *p* = 0.030), and the presence of pathological lymph node metastasis (HR = 4.73, *p* = 0.030) were correlated with significantly higher risks of death (Table 2). In a multivariate analysis, the NLR (HR = 4.62, *p* = 0.030), CRP (HR = 10.80, *p* = 0.045), and pathological lymph node metastasis (HR = 12.35, *p* = 0.009) were also found to be significantly associated with OS [Table 2]. A Kaplan-Meier analysis and log-rank test further revealed that a high NLR was correlated with a significantly lower rate of OS, in comparison to a low NLR (*p* = 0.018; Fig. 2). However, the association between the NLR and disease progression was not statistically significant (*p* = 0.137).

**Table 2:** Univariate and multivariate analyses of factors for the OS

		Median OS	Univariate analysis			Multivariate analysis		
	n	(mean ± SD)	HR	95%CI	*p*-value	HR	95%CI	*p*-value
Age								
<65 years	32	2122 (2221.6 ± 1725.5)	1	0.50-4.73	0.453			
≥65 years	42	718 (1071.4 ± 1003.6)	1.54					
Gender								
Female	16	767 (1891.9 ± 1536.9)	1	0.50-12.02	0.272			
Male	58	980 (1479.6 ± 13743.5)	2.44					
ECOG-PS								
0	68	1286 (1673.7 ± 1413.1)	1	0.7021.22	0.121			
≥1	6	302 (379.3 ± 319.4)	3.86					
NLR								
<2.38	51	1250.5 (1576.6 ± 1375.0)	1	1.54-15.23	0.007	1	1.16-18.34	0.030
≥2.38	23	797.5 (1551.3 ± 1487.5)	4.84			4.62		
CPR								
<0.08	23	1427 (1461.3 ± 1091.4)	1	1.24-81.27	0.030	1	1.06-110.29	0.045
≥0.08	51	772 (1617.2 ± 1533.2)	10.06			10.8		
LDH								
<158	20	1286 (1464.7 ± 1205.3)	1	0.36-4.47	0.712			
≥158	54	808 (1607.3 ± 1565.7)	1.27					
Neoadjuvant Chemotherapy								
No	64	1244 (1535.9 ± 1339.8)	1	0.15-4.10	0.772			
Yes	10	331 (1779.3 ± 2228.4)	0.78					
Clinical T stage								
≤2	38	980 (1828.6 ± 1722.9)	1	0.39-3.47	0.781			
≥3	36	818 (1294.5 ± 1114.1)	1.67					
Clinical Lymph Node Metastasis								
No	70	818 (1566.6 ± 1492.3)	1	0.11-11.57	0.921			
Yes	4	1671 (1607.3 ± 615.3)	1.13					
Sugical Margin								
Negative	68	11271.5 (1616.1 ± 1431.6)	1	0.85-7.74	0.095			
Positive	6	707 (1287.8 ± 2064.0)	2.56					
Adjuvant chemotherapy								
No	49	722 (1189.7 ± 1200.8)	1	0.48-4.52	0.493			
Yes	25	2154 (2311.7 ± 1624.5)	1.48					
Pathological T stage								
≤2	53	1112 (1586.9 ± 1466.4)	1	0.32-3.52	0.914			
≥3	21	982 (1437.4 ± 1603.1)	1.07					
Pathological Lymph Node Metastasis								
No	64	1244 (1609.8 ± 1466.4)	1	1.17-19.17	0.030	1	1.85-82.47	0.009
Yes	10	718 (1305.9 ± 1523.6)	4.73			12.35		

OS: overall survival, ECOG-PS: Eastern Cooperative Oncology Group performance status, NLR: neutrophil-to-lymphocyte ration

CPR: C-reactive protein, LHD: lactate dehydrogenase

**Table 3:** The association between the NLR and the prognosis of urothelial carcinoma

Study	Year	Number of patients	Cut-off value	Prognosis (high vs low NLR)	Hazard ratio (low NLR was set as 1)
Bladder					
Gondo et al. [28]	2012	189	2.5	not mentioned (OS)	1.946, p==0.0015
Demirtas et al. [2]	2013	201	2.5	no significant differences	N/A
Krane et al. [37]	2013	68	2.5	23 vs 36 months (CSS)	2.25
Hermanns et al. [26]	2014	424	3	43 % vs 64 % (OS)	1.67, p < 0.001
Temraz et al. [38]	2014	68	2.87	2.7 vs 6.0 years (OS)	1.88
Mano et al. [33]	2014	107 (NMIBC)	2.41		3.74, p = 0.004
Viers et al. [39]	2014	unknown	unknown		1.03, p = 0.01
Kayner et al. [40]	2014	291	2.50	NMIBC vs MIBC	p = 0.028
Current study	2014	74	2.38	52.6 vs 51.7 months (OS)	4.84, p = 0.007
Upper urinary tract					
Azuma et al. [41]	2013	137	2.5	29.4 % vs 81.3 % (OS)	6.14, p < 0.0001
Dalpiaz et al. [9]	2013	202	2.7	27 vs 44.5 months (OS)	3.073, p < 0.001

NLR: neutrophil-to-lymphocyte ration, NMIBC: non-muscle-invasive bladder cancer, MIBC: muscle-invasive bladder cancer, OS: overall survival, CSS: cancer-specific survival

### Tumor infiltrating lymphocytes and tumor grade/stage

**Fig. 3:**
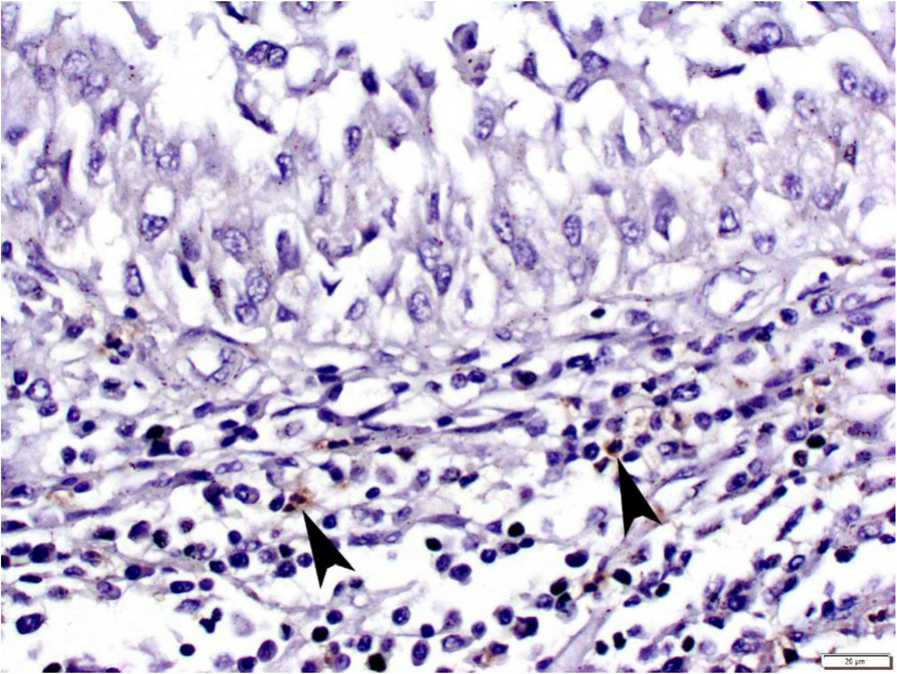
Immunohistochemistry of CD66b in the bladder TMA. Occasional CD66b-positive neutrophils (arrowheads) are seen in the stromal tissue. Original magnification, x400

**Fig. 4:**
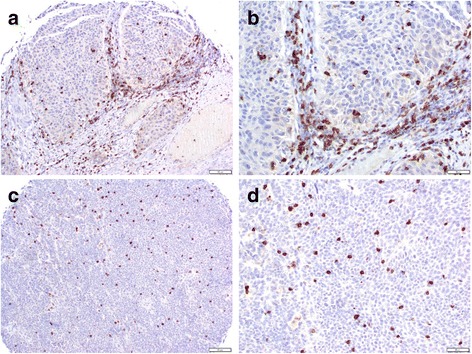
Immunohistochemistry of CD8 in the bladder TMA. Infiltrating CD8-positive lymphocytes are present mainly in the stromal (**a**, original magnification x100; **b**, original magnification x200) or intratumoral (**c**, original magnification x100; **d**, original magnification x200) compartments

CD66b-positve cells were only observed in a few cases (Fig. 3). Therefore, we analyzed the relationship between the number of tumor infiltrating CD8-positive lymphocytes (Fig. 4) and the tumor grade or stage. The number of CD8-positive lymphocytes was significantly increased in high-grade (mean ± SD: 29.4 ± 23.5) and muscle-invasive (23.8 ± 22.4) tumors, in comparison to low-grade (15.1 ± 17.1; p = 0.001) and non-muscle-invasive (15.1 ± 17.8; p = 0.012) tumors. The number of CD8-positive cells did not differ significantly in benign urothelial tissues (mean ± SD: 21.6 ± 12.7) and urothelial carcinomas (19.1 ± 20.0) (p = 0.291).

## Discussion

There is increasing evidence to show that the presence of systemic inflammation is correlated with poorer cancer-specific survival in several solid tumors, such as colorectal carcinoma [6, 15–20]. Moreover, non-steroidal anti-inflammatory medications have been suggested to reduce the risk of developing bladder cancer, which implies a critical correlation between inflammation and bladder tumorigenesis [16, 21]. This study revealed systemic inflammation including CRP was an independent risk factor to estimate the prognosis. The presence of an inflammatory response can be determined by both the expression of CRP and an elevation in the NLR [4, 6, 22]. The latter has indeed been shown to be associated with a poorer prognosis in patients with some solid tumors [4, 6, 11, 23, 24]. The proposed mechanisms include increasing the supply of growth factors, survival factors, pro-antigenic factors, extracellular matrix-modifying enzymes that can facilitate invasion and metastasis, and inductive signals that may lead to epithelial-to-mesenchymal transition [25, 26].. The interaction between the tumor and the immune system of the host not only promotes tumor cell proliferation and metastasis but also activates the inflammatory cascade in the host, which leads to the further deterioration of the general condition of cancer patients [27]. The NLR, as an independent parameter, has been shown to be significantly correlated with serum CRP levels [28]. However, another study [2], found that the NLR was not a powerful predictor of survival. Thus, there is a biological rationale for using the NLR, as a measure of systemic host response when evaluating the association between inflammation and cancer outcomes [26].

It has been proposed that the NLR can be used to estimate the magnitude of systemic inflammation in cancer patients [5, 29–31]. It can easily be calculated from routine CBCs with differentials [26]. CBCs are usually determined in the clinical check-ups, thus it is possible to apply the NLR to all patients, both preoperatively and postoperatively. Thus, the NLR is a useful tool when considering additional therapy after radical cystectomy. We also attempted to investigate the numbers of CD66b-positive neutrophils and CD8-positive lymphocytes in separate sets of bladder cancer tissues. An immunohistochemical analysis showed that CD8-positive cells were present in the majority of cases, whereas CD66b-positve cells were seen only in a few cases. Higher numbers of CD8-positive lymphocytes were strongly correlated with a higher tumor grade or stage. In a previous immunohistochemical study involving 56 cystectomy cases, a high CD8 density (defined as the presence of ≥60 intra-tumoral CD8-positive cells per high-power field) was observed in 10 of 45 (22 %) muscle-invasive tumors, while it was not observed in any of 11 non-muscle-invasive tumors [32]. In contrast, although all of the cystectomy cases in which the NLR was assessed in the present study exhibited muscle-invasive tumors, we found no significant difference in the NLRs of patients with different pT or pN stages. Thus, there appeared to be no strong correlation between the number of CD8-positive lymphocytes in the tissue specimens and the NLR in blood in bladder cancer patients. Nevertheless, in a previous study using tissue specimens, a high CD8 density was only associated with a favorable prognosis in ≥ pT1 tumors, which supports the prognostic significance of the NLR via CBCs [32].

The present study showed that the NLR was an independent prognostic factor in bladder cancer patients who underwent radical cystectomy. Tumor characteristics are known to assist in the prediction of the risk of disease recurrence and progression [33]. The European Organization for Research and Treatment of Cancer (EORTC) risk table has been commonly used to predict progression [33–36]. In order to further improve the prediction, new biomarkers are needed. In bladder cancer, several studies have shown the NLR to be a prognostic factor [2, 26, 28, 37–39]. On the other hand, the association between the NLR and tumor progression remains controversial (Table 3). These studies showed that a higher NLR was correlated with a worse prognosis in patients with bladder cancer, while others indicated that the NLR was not recognized to be correlated with OS [2, 26, 28, 37, 38]. In patients with non-muscle-invasive bladder cancer, an NLR of >2.41 tended to be correlated with recurrence and progression after transurethral resection of bladder tumors [33]. In the present study, a high NLR was found to be a risk factor for death in patients with invasive bladder cancer who underwent radical cystectomy.

The AUROC determined the cut-off value of the NLR to be 2.38 in the present study. Kayner et al. reported that MIBC showed a higher NLR compared with NMIBC, and the cut-off point was set as 2.50 in their study [40]. Several studies in advanced pancreatic cancer showed NLR cut-off values of approximately 5 [5]. In intrahepatic cholangiocarcinoma [6], and in patients with liver metastasis from colorectal carcinoma [25], the NLR cut-off value was also set as 5. In urologic cancers, NLR cut-off values of approximately 5 (prostate cancer) or from 2 to 5 (renal cell carcinoma) have been used [41]. In urothelial carcinoma, NLR cut-off values of 2.41-3.0 have been reported [2, 26, 37, 38, 40, 42]. Our cut-off point for the NLR was thus somewhat lower than the values of previous reports.

The present study is associated with several limitations. First, this is a retrospective study, which may have led selection bias. Second, we did not perform mechanistic experiments to determine the roles of neutrophils and/or lymphocytes in bladder cancer progression. Nonetheless, the current results support the findings of previous studies which indicate correlations between NLR/inflammation and the clinical outcome of patients with muscle-invasive bladder cancer. The NLR may thus be useful in the prediction of prognosis in bladder cancer patients after radical cystectomy.

## Conclusions

The current study demonstrated the usefulness of the NLR in the prediction of OS in patients with advanced stage bladder cancer. The NLR was thus found to be an independent prognostic marker for predicting the prognosis. The preoperative NLR predicted prognosis in patients who underwent radical cystectomy and might therefore function as a reliable biomarker for invasive bladder cancer.

## Availability of supporting data

Due to ethical restrictions, the raw data underlying this paper are available upon request to the corresponding author.

## References

[1] Jemal A, Siegel R, Xu J, Ward E (2010). Cancer statistics, 2010. CA Cancer J Clin.

[2] Demirtas A, Sabur V, Akinsal EC, Demirci D, Ekmekcioglu O, Gulmez I, Tatlisen A (2013). Can neutrophil-lymphocyte ratio and lymph node density be used as prognostic factors in patients undergoing radical cystectomy?. Sci World J.

[3] Lucca I, de Martino M, Klatte T, Shariat SF (2015). Novel Biomarkers to Predict Response and Prognosis in Localized Bladder Cancer. Urol Clin North Am.

[4] Zahorec R (2001). Ratio of neutrophil to lymphocyte counts--rapid and simple parameter of systemic inflammation and stress in critically ill. Bratisl Lek Listy.

[5] Xue P, Kanai M, Mori Y, Nishimura T, Uza N, Kodama Y, Kawaguchi Y, Takaori K, Matsumoto S, Uemoto S (2014). Neutrophil-to-lymphocyte ratio for predicting palliative chemotherapy outcomes in advanced pancreatic cancer patients. Cancer medicine.

[6] Gomez D, Morris-Stiff G, Toogood GJ, Lodge JP, Prasad KR (2008). Impact of systemic inflammation on outcome following resection for intrahepatic cholangiocarcinoma. J Surg Oncol.

[7] Chua W, Charles KA, Baracos VE, Clarke SJ (2011). Neutrophil/lymphocyte ratio predicts chemotherapy outcomes in patients with advanced colorectal cancer. Br J Cancer.

[8] Azab B, Bhatt VR, Phookan J, Murukutla S, Kohn N, Terjanian T, Widmann WD (2012). Usefulness of the neutrophil-to-lymphocyte ratio in predicting short- and long-term mortality in breast cancer patients. Ann Surg Oncol.

[9] Dalpiaz O, Pichler M, Mannweiler S, Martin Hernandez JM, Stojakovic T, Pummer K, Zigeuner R, Hutterer GC (2014). Validation of the pretreatment derived neutrophil-lymphocyte ratio as a prognostic factor in a European cohort of patients with upper tract urothelial carcinoma. Br J Cancer.

[10] Jung MR, Park YK, Jeong O, Seon JW, Ryu SY, Kim DY, Kim YJ (2011). Elevated preoperative neutrophil to lymphocyte ratio predicts poor survival following resection in late stage gastric cancer. J Surg Oncol.

[11] Walsh SR, Cook EJ, Goulder F, Justin TA, Keeling NJ (2005). Neutrophil-lymphocyte ratio as a prognostic factor in colorectal cancer. J Surg Oncol.

[12] Ohno Y, Nakashima J, Ohori M, Hatano T, Tachibana M (2010). Pretreatment neutrophil-to-lymphocyte ratio as an independent predictor of recurrence in patients with nonmetastatic renal cell carcinoma. J Urol.

[13] Rosenberg L, Lawlor GO, Zenlea T, Goldsmith JD, Gifford A, Falchuk KR, Wolf JL, Cheifetz AS, Robson SC, Moss AC (2013). Predictors of endoscopic inflammation in patients with ulcerative colitis in clinical remission. Inflamm Bowel Dis.

[14] Kawahara T, Kashiwagi E, Ide H, Li Y, Zheng Y, Miyamoto Y, Netto GJ, Ishiguro H, Miyamoto H (2015). Cyclosporine A and tacrolimus inhibit bladder cancer growth through down-regulation of NFATc1. Oncotarget.

[15] Kawahara T, Ishiguro H, Hoshino K, Teranishi J, Miyoshi Y, Kubota Y, Uemura H (2010). Analysis of NSAID-activated gene 1 expression in prostate cancer. Urol Int.

[16] Ishiguro H, Kawahara T (2014). Nonsteroidal anti-inflammatory drugs and prostatic diseases. BioMed research international.

[17] Coussens LM, Werb Z (2002). Inflammation and cancer. Nature.

[18] Gunter MJ, Stolzenberg-Solomon R, Cross AJ, Leitzmann MF, Weinstein S, Wood RJ, Virtamo J, Taylor PR, Albanes D, Sinha R (2006). A prospective study of serum C-reactive protein and colorectal cancer risk in men. Cancer Res.

[19] Zhang K, Kaufman RJ (2008). From endoplasmic-reticulum stress to the inflammatory response. Nature.

[20] Kawahara T, Ito H, Terao H, Yoshida M, Ogawa T, Uemura H, Kubota Y, Matsuzaki J (2012). Ureteroscopy assisted retrograde nephrostomy: a new technique for percutaneous nephrolithotomy (PCNL). BJU Int.

[21] Castelao JE, Yuan JM, Gago-Dominguez M, Yu MC, Ross RK (2000). Non-steroidal anti-inflammatory drugs and bladder cancer prevention. Br J Cancer.

[22] McMillan DC, Canna K, McArdle CS (2003). Systemic inflammatory response predicts survival following curative resection of colorectal cancer. Br J Surg.

[23] Duffy BK, Gurm HS, Rajagopal V, Gupta R, Ellis SG, Bhatt DL (2006). Usefulness of an elevated neutrophil to lymphocyte ratio in predicting long-term mortality after percutaneous coronary intervention. Am J Cardiol.

[24] Halazun KJ, Aldoori A, Malik HZ, Al-Mukhtar A, Prasad KR, Toogood GJ, Lodge JP (2008). Elevated preoperative neutrophil to lymphocyte ratio predicts survival following hepatic resection for colorectal liver metastases. European journal of surgical oncology : the journal of the European Society of Surgical Oncology and the British Association of Surgical Oncology.

[25] Hanahan D, Weinberg RA (2011). Hallmarks of cancer: the next generation. Cell.

[26] Hermanns T, Bhindi B, Wei Y, Yu J, Noon AP, Richard PO, Bhatt JR, Almatar A, Jewett MA, Fleshner NE (2014). Pre-treatment neutrophil-to-lymphocyte ratio as predictor of adverse outcomes in patients undergoing radical cystectomy for urothelial carcinoma of the bladder. Br J Cancer.

[27] Mantovani A, Allavena P, Sica A, Balkwill F (2008). Cancer-related inflammation. Nature.

[28] Gondo T, Nakashima J, Ohno Y, Choichiro O, Horiguchi Y, Namiki K, Yoshioka K, Ohori M, Hatano T, Tachibana M (2012). Prognostic value of neutrophil-to-lymphocyte ratio and establishment of novel preoperative risk stratification model in bladder cancer patients treated with radical cystectomy. Urology.

[29] Stotz M, Gerger A, Eisner F, Szkandera J, Loibner H, Ress AL, Kornprat P, AlZoughbi W, Seggewies FS, Lackner C (2013). Increased neutrophil-lymphocyte ratio is a poor prognostic factor in patients with primary operable and inoperable pancreatic cancer. Br J Cancer.

[30] Smith RA, Bosonnet L, Raraty M, Sutton R, Neoptolemos JP, Campbell F, Ghaneh P (2009). Preoperative platelet-lymphocyte ratio is an independent significant prognostic marker in resected pancreatic ductal adenocarcinoma. Am J Surg.

[31] Proctor MJ, Morrison DS, Talwar D, Balmer SM, O’Reilly DS, Foulis AK, Horgan PG, McMillan DC (2011). An inflammation-based prognostic score (mGPS) predicts cancer survival independent of tumour site: a Glasgow Inflammation Outcome Study. Br J Cancer.

[32] Faraj SF, Munari E, Guner G, Taube J, Anders R, Hicks J, Meeker A, Schoenberg M, Bivalacqua T, Drake C (2015). Assessment of tumoral PD-L1 expression and intratumoral CD8+ T cells in urothelial carcinoma. Urology.

[33] Mano R, Baniel J, Shoshany O, Margel D, Bar-On T, Nativ O, Rubinstein J, Halachmi S. Neutrophil-to-lymphocyte ratio predicts progression and recurrence of non-muscle-invasive bladder cancer. Urologic oncology. 2015;33(2):67. e1-7. 10.1016/j.urolonc.2014.06.010 25060672

[34] Babjuk M, Burger M, Zigeuner R, Shariat SF, van Rhijn BW, Comperat E, Sylvester RJ, Kaasinen E, Bohle A, Palou Redorta J (2013). EAU guidelines on non-muscle-invasive urothelial carcinoma of the bladder: update 2013. Eur Urol.

[35] Brausi M, Witjes JA, Lamm D, Persad R, Palou J, Colombel M, Buckley R, Soloway M, Akaza H, Bohle A (2011). A review of current guidelines and best practice recommendations for the management of nonmuscle invasive bladder cancer by the International Bladder Cancer Group. J Urol.

[36] Babjuk M, Oosterlinck W, Sylvester R, Kaasinen E, Bohle A, Palou-Redorta J, European Association of U (2008). EAU guidelines on non-muscle-invasive urothelial carcinoma of the bladder. Eur Urol.

[37] Krane LS, Richards KA, Kader AK, Davis R, Balaji KC, Hemal AK (2013). Preoperative neutrophil/lymphocyte ratio predicts overall survival and extravesical disease in patients undergoing radical cystectomy. Journal of endourology/Endourological Society.

[38] Temraz S, Mukherji D, Farhat ZA, Nasr R, Charafeddine M, Shahait M, Wehbe MR, Ghaida RA, Gheida IA, Shamseddine A (2014). Preoperative lymphocyte-to-monocyte ratio predicts clinical outcome in patients undergoing radical cystectomy for transitional cell carcinoma of the bladder: a retrospective analysis. BMC Urol.

[39] Viers BR, Boorjian SA, Frank I, Tarrell RF, Thapa P, Karnes RJ, Thompson RH, Tollefson MK: Pretreatment Neutrophil-to-Lymphocyte Ratio Is Associated with Advanced Pathologic Tumor Stage and Increased Cancer-specific Mortality Among Patients with Urothelial Carcinoma of the Bladder Undergoing Radical Cystectomy. Eur Urol. 2014;66(6):1157-64. 10.1016/j.eururo.2014.02.042 24630414

[40] Kaynar M, Yildirim ME, Badem H, Cavis M, Tekinarslan E, Istanbulluoglu MO, Karatas OF, Cimentepe E (2014). Bladder cancer invasion predictability based on preoperative neutrophil-lymphocyte ratio. Tumour Biol.

[41] Wei Y, Jiang YZ, Qian WH (2014). Prognostic role of NLR in urinary cancers: a meta-analysis. PLoS One.

[42] Azuma T, Matayoshi Y, Odani K, Sato Y, Sato Y, Nagase Y, Oshi M (2013). Preoperative neutrophil-lymphocyte ratio as an independent prognostic marker for patients with upper urinary tract urothelial carcinoma. Clin Genitourin Cancer.

